# A comprehensive lettuce variation map reveals the impact of structural variations in agronomic traits

**DOI:** 10.1186/s12864-023-09739-x

**Published:** 2023-11-02

**Authors:** Zhaowu Zhang, Rob van Treuren, Ting Yang, Yulan Hu, Wenhui Zhou, Huan Liu, Tong Wei

**Affiliations:** 1 College of Life Sciences, University of Chinese Academy of Sciences, Beijing, 100049 China; 2 State Key Laboratory of Agricultural Genomics, BGI Research, Shenzhen, 518083 China; 3 Centre for Genetic Resources, the Netherlands, Wageningen University & Research, Wageningen, the Netherlands

**Keywords:** Cultivated lettuce, Structural variations, Genome-wide association analysis

## Abstract

**Background:**

As an important vegetable crop, cultivated lettuce is grown worldwide and a great variety of agronomic traits have been preserved within germplasm collections. The mechanisms underlying these phenotypic variations remain to be elucidated in association with sequence variations. Compared with single nucleotide polymorphisms, structural variations (SVs) that have more impacts on gene functions remain largely uncharacterized in the lettuce genome.

**Results:**

Here, we produced a comprehensive SV set for 333 wild and cultivated lettuce accessions. Comparison of SV frequencies showed that the SVs prevalent in *L. sativa* affected the genes enriched in carbohydrate derivative catabolic and secondary metabolic processes. Genome-wide association analysis of seven agronomic traits uncovered potentially causal SVs associated with seed coat color and leaf anthocyanin content.

**Conclusion:**

Our work characterized a great abundance of SVs in the lettuce genome, and provides a valuable genomic resource for future lettuce breeding.

**Supplementary Information:**

The online version contains supplementary material available at 10.1186/s12864-023-09739-x.

## Background

Sequence variations among individuals form the basis of phenotypic variations in crop species [1]. It is of great importance to understand the distribution and impacts of these variants in germplasm collections in order to utilize them in breeding programs [2–4]. Sequence variants are classified based on their sizes into single-nucleotide polymorphisms (SNPs), insertion/deletions (indels), and structural variations (SVs) [5, 6]. Indels consist insertions and deletions smaller than 50 bp, while SV is generally defined as a genomic variants over 50 bp, including deletions (DELs), insertions (INSs), duplications (DUPs), inversions (INVs) and chromosomal translocations (TRAs) [7]. Compared with SNPs and indels, SVs often have greater impacts on genome architectures and gene functions [8–10]. SV identification is the key to understand these important variants, but it has been hampered by the complexity and relatively large size [11, 12].

Lettuce is one of the most valuable vegetable crops worldwide, together with chicory produces more than 27 million tons in 2020 worldwide [13]. Cultivated lettuce is a primary ingredient in green salad, and a great variety of cultivars have been developed to meet consumers’ needs. Various agronomic traits, such as leaf shape, color, and texture, have been constantly selected by breeders [14, 15]. Understanding the underlying genetic mechanism would greatly facilitate the molecular breeding of favorable traits in cultivated lettuce [16]. In a previous study, 445 *Lactuca* accessions, including the major lettuce cultivated types and wild relative species, were sequenced and a comprehensive variome was developed, from which the population structure and domestication history of cultivated lettuce were revealed [17]. Genome-wide association analyses with SNPs Identified genetic loci related to important agronomic traits. However, the impacts of SVs on the lettuce genome and agronomic traits have not been fully characterized.

Here, we generated a set of 3,693,607 SVs using an optimized pipeline from 333 cultivated and wild lettuce (*L. sativa* and *L. serriola*) accessions. Comparison of SV frequencies indicates that SVs have potential impact on carbohydrate and secondary metabolism in cultivated lettuce. Genome-wide association analysis using SVs identified candidate genes related to seed coat color and leaf anthocyanin content among other agronomic traits. This study improves our understanding of SVs in the lettuce genome, and provides a valuable resource for lettuce research and breeding in the future.

## Results

### Construction of a comprehensive SV set for cultivated and wild lettuce

**Fig. 1:**
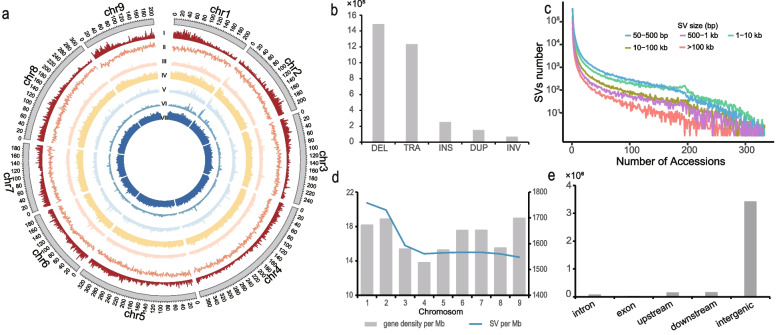
SVs in 133 *L. sativa* and 200 *L. serriola* accessions. **a** The distribution of SVs along the lettuce genome. Circos plot from outer to inter tracks shows, (I) gene density in a 1-Mb sliding window, (II) GC content, (III-VII) SV count in the 1-Mb window for various SV types, including DEL (III), INS (IV), TRA (V), INV (VI), and DUP (VII). **b** The number of different SV types. **c** Detection frequencies of SVs with different sizes. **d** The density of SVs and gene on each chromosome (count per Mb). **e** The number of SVs from different generic regions

To construct a comprehensive SV set, we used whole genome resequencing (WGS) data of 333 wild and cultivated lettuce accessions from a previous work, and three SV caller. Manta [18], Delly [19], and Breakdancer [20] for SV identification SVs. we optimized the parameters for SV combination and generated a total of 3,693,607 SVs from 133 *L. sativa* and 200 *L. serriola* accessions (Fig. 1a, Table S1). This SV set was composed of 1,736,147 DELs, 263,620 INSs, 168,774 DUPs, 74,429 INVs, and 1,450,637 TRAs (Fig. 1b, Table S2). The SVs ranging from 50–500 bp and 1–10 kb accounted for the largest two proportions of total SVs, 36.81% and 33.66% respectively. SVs of 10–100 kb and 0.5–1 kb were 14.88% and 9.51%, respectively, and SVs above 100 kb were relatively rare (5.14%; Fig. 1c). The lengths of DELs had a similar distribution as the whole SV set with those of 50–500 bp and 1–10 kb formed the largest two proportions (37.25% and 35.41%, respectively). Those over 100 kb accounted for 2.60% of total DELs, which were even rarer than in the total SVs (Fig. S1a). The other three SV types exhibited different distribution patterns. Nearly all the detected INSs were from 50 to 500 bp, most likely caused by difficulty in detecting long insertions from short reads. The most abundant DUPs were from 1–10 kb and 10–100 kb (39.62% and 27.91%, respectively), and nearly half of INVs were above 100 kb (49.82%) (Fig. 1c, Fig. S1b-d). The variation in length distribution among different SV types reflect that they may have different influence on the lettuce genome.

As shown in Fig. 1d, SVs were unevenly distributed across the lettuce genome, with the average SV numbers per Mb ranging from 1757.87 on chromosome 1 to 1547.74 on chromosome 9. In the lettuce genome, 88.25% SVs reside in intergenic region and the rest were from protein-coding genes (Fig. 1e). Among those from the genic regions, 0.85% were overlapped with exons, 3.21% were overlapped with introns, 4.20% were overlapped with 1-kb upstream regions, and 3.89% were overlapped with 1-kb downstream regions.

### SV impact in cultivated lettuce

To investigate the impact of SVs on gene functions, we annotated the SVs using SnpEff and found that 38,255 out of 38,901 annotated genes were affected by at least one SV. Among more than 3.6 million SVs, 223,840 were predicted with high impacts by disrupting gene functions, 75,370 SVs with moderate impacts that cause amino acid change, 986,174 SVs with low impacts that cause no changes in protein sequences, and 2,548,899 SVs were classified as modifier. The high-impact SVs included 138,311 DELs, 454 INSs, 9,696 DUPs, 6,951 INVs, and 68,428 TRAs, which affected 38,177, 442, 3,820, 1,867 and 12,346 genes, respectively. As the majority of DELs were from 50–500 bp and 1–10 kb, the genes affected by DELs were most likely disrupted.

**Fig. 2:**
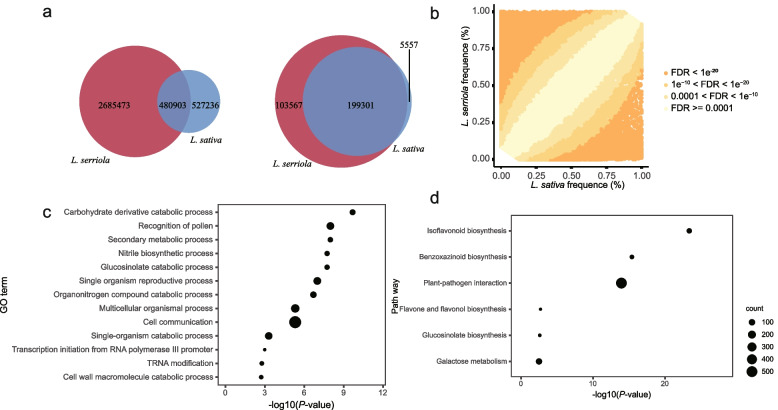
SV divergence between *L. sativa* and *L. serriola*. **a** Venn diagram of SVs from *L. sativa* and *L. serriola*. In the left panel is from whole SV set, and the right panel from the filtered set. **b** Scatter plot showing SV frequencies in the *L. sativa* and *L. serriola* groups. Color from orange to light yellow represents FDR-adjusted *P* values from low to high. **c** GO terms enriched in genes affected by the SVs with significantly higher frequencies in *L. sativa* than in *L. serriola*. **d** Pathway enriched in genes affected by the SVs with significantly higher frequencies in *L. sativa* than in *L. serriola*

Among 3,693,607 SVs, 480,903 were shared between *L. sativa* and *L. serriola*, which account for 47.70% of those identified in *L. sativa* and 15.19% in *L. serriola* (Fig. 2a). The length, number and SV positions relative to protein-coding genes distribution of SVs in *L. sativa* and *L. serriola* were similar (Figs. S2b-c and S3b-c). SVs were unevenly distributed in the genomes of *L. sativa* and *L. serriola*, with the largest difference observed on chromosome 6. The SVs density in *L. serriola* was 3.84 times that of *L. sativa* on chromosome 6 (Figs. S2d and S3d).

A filtered set of 308,420 SVs was produced using the minor allele frequency of < 0.05 to obtain common alleles in the population. In the filtered SV set, 199,301 were shared between *L. sativa* and *L. serriola*, which account for 97.29% of common SVs in *L. sativa* and 65.80% in *L. serriola* (Fig. 2a). These results agreed with *L. serriola* as the progenitor and primary gene pool of *L. sativa *[21].

To evaluate which SVs were preferably distributed in *L. sativa*, we compared the SV frequencies between *L. sativa* and *L. serriola*. A total of 224,041 common SVs showed significant differences (FDR < 0.0001), among which 43,711 displayed higher frequencies in *L. sativa*, while 180,330 with higher frequencies in *L. serriola* (Fig. 2b, Table S3). A total of 6,502 genes were affected by *L. sativa-*predominant SVs, which were enriched with carbohydrate derivative catabolic and secondary metabolic process in *L. sativa* (Fig. 2c, Table S4). Another enrichment analysis of metabolic pathways showed that isoflavonoid biosynthesis, benzoxazinoid biosynthesis, plant-pathogen interaction, flavone and flavonol biosynthesis, glucosinolate biosynthesis, and galactose metabolism were enriched in the same 6,502 SV-affected genes (Fig. 2d, Table S5). Considering the majority of common SVs consisting of DELs that may disrupt gene function, the enrichment of these pathways suggests some of the related genes were likely to be selected against during lettuce domestication and improvement.

### Population structure revealed by SVs

**Fig. 3:**
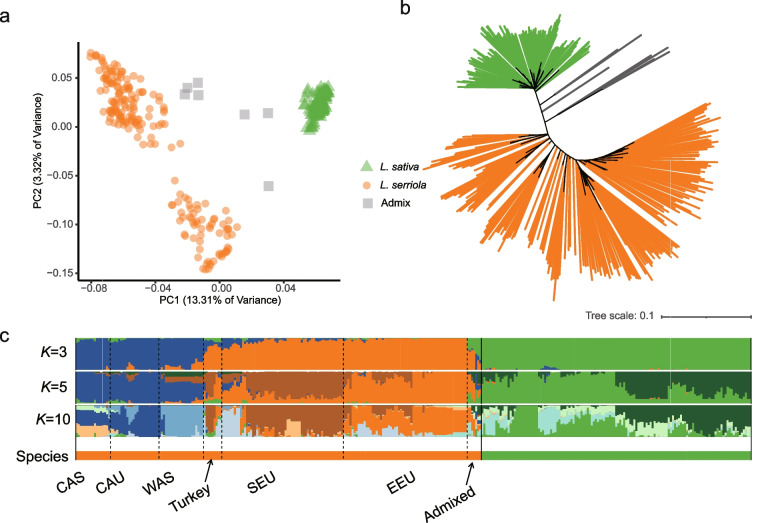
Population structure in 333 *L. sativa* and *L. serriola* accessions. **a** Principal component analysis (PCA) using the filtered SVs, in which green color represents *L. sativa,* yellow for *L. serriola* and gray for admixed samples between *L. sativa* and *L. serriola*. **b** Neighbor-joining tree with branch colors representing *L. sativa* (green), *L. serriola* (orange), and admixed samples (grey). **c** Model-based clustering analysis with different numbers of ancestry kinship (*K* = 3,5 or 10). Species are indicated in the colored bar at the bottom, with the green color for *L. sativa* and orange for *L. serriola*. Geographic groups of *L. serriola* are indicated in text from central Asia (CAS), the Caucasus (CAU), western Asia (WAS), southern Europe (SEU), and eastern Europe (EEU). Turkish samples and the admixed ones from *L. sativa* and *L. serriola* are indicated by arrows

To determine whether SVs represent the genetic architecture of this germplasm collection, we carried out population analyses using the filtered SV set. The principal component analysis (PCA) showed that the 333 investigated samples were separated into three distinct groups, one of *L. sativa* accessions and two of the *L. serriola* ones from Asia and Europe (Fig. 3a). In the neighbor-joining tree, all the *L. sativa* accessions formed a single clade while the *L. serriola* samples formed several clusters from various geographical origins (Fig. 3b). The cross-validation value met the minimum when assuming five ancestries (Fig. 3c, Fig. S5), with two sub-groups in *L. sativa* (Butterhead and other cultivars) and three sub-groups in *L. serriola* (Asian, southern and eastern European) (Fig. S4). A detailed intra-specific structure was further revealed when assuming ten ancestries, in which *L. serriola* were divided into five major geographic groups from central Asia, the Caucasus, western Asia, southern and eastern Europe. The admixed samples between *L. sativa* and *L. serriola*, and *L. serriola* from Turkey showed admixed genetic compositions (Fig. S6).

To determine the differences in SV between geographic group, we counted the specific SV of different geographical groups, and we found that the Caucasus group had the highest proportion of specific SV (Table S6), which was consistent with the highest nucleotide diversity in the Caucasian group [17]. Pathway analyses showed that the genes involved in flavone, isoflavonoid and flavonol biosynthesis were enriched in those affected by Caucasus-specific SVs, and the gene involved in plant-pathogen interaction enriched in those affected by the SVs specific to the western Asian and eastern European groups (Table S7). These results revealed the population structure of *L. sativa* and *L. serriola*, which agreed with the previous results based on SNPs [17].

### Association of SVs with agronomic traits

**Fig. 4:**
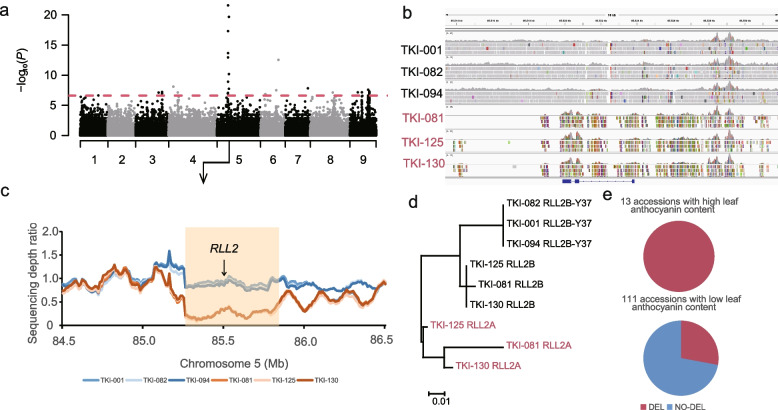
Genome-wide association study (GWAS) of leaf anthocyanin content in *L. sativa* using filtered SVs. **a** Manhattan plots of GWAS across nine lettuce chromosomes. **b** Read alignment on the genomic regions flanking the *RLL2* gene shown in the IGV interface. The accessions with high anthocyanin content were colored in red. The *RLL2* gene structure is shown at the bottom. **c** Sequencing depth ratio within the association region in six representative accessions. The 600-kb deletion is indicated by the orange box, and *RLL2* is indicated by the arrow. The orange and blue lines indicate the relative sequencing depth (read coverage within a 100-kb window with 10-kb step divided by the average depth across the genome) in three representative accessions with high and low anthocyanin content, respectively. **d** A Maximum-likelihood phylogenetic tree of RLL2 protein sequences from six representative accessions. The accessions with high anthocyanin content were colored in red. **e** The frequency of the 600-kb deletion in *L. sativa* accessions with high and low anthocyanin content

To estimate the impacts of SVs on phenotypic variations in cultivated lettuce, we conducted a genome-wide association study (GWAS) on seven agronomic traits, including seed coat color, flower anthocyanin presence, leaf margin undulation, leaf anthocyanin content, seedling cotyledon shape, leaf venation and leaf morphology. A total of ten signals were found associated with six traits (Fig. S7, Table S8), which mostly agreed with previous GWAS results using SNPs [17]. For example, a 1.63-Mb region from 84.53 to 86.16 Mb region on chromosome 5 was found in association with leaf anthocyanin content. This region contains five SVs, 29 SNPs, and 44 indels (Fig. 4a). The associated variants were linked with *LG5_40441* near 85.52 Mb, which encode RLL2 transcription factor controlling leaf color as previously reported [15]. Although no disruptive variants were found within the coding region, we noticed a deletion surrounding *LG5_40441* as illustrated in read alignment (Fig. 4b). We calculated the sequencing depth within a 100-kb window with a 10-kb step from 84 to 87 Mb on chromosome 5, and identified a deletion from 85.24 to 85.84 Mb containing the *RLL2* gene (Fig. 4c). This 600-kb deletion was detected in all 13 accessions with high anthocyanin content and 31 out of 111 ones with low content (Fig. 4e). Despite the 600-kb deletion found in those with high anthocyanin content, the read alignments on *LG5_40441* suggests the presence of a functional *RLL2A* gene (Fig. 4b). We therefore assembled contigs for six representative samples, and identified the *RLL2A* coding sequence in those with high anthocyanin content but not in low-content ones. *RLL2B* was also identified along with *RLL2A* from high-content accessions, while *RLL2B-Y37* was the only allele found in those with low content (Data S1). Phylogenetic analysis showed that the *RLL2B* alleles was in the same clade as *RLL2B-Y37*, while the *RLL2A* alleles formed a distinct clade (Fig. 4d). Our results suggest a possible presence-absence variation carrying different *RLL2* alleles leads to the phenotypic variation in leaf anthocyanin content.

An uncharacterized trait was explored here in lettuce seeds, for which 52 *L. sativa* accessions were recorded with black seed coat and 81 with white seed coat in our collection (Fig. S8). PCA analyses were performed using filtered SNPs, Indels, and SVs in *L. sativa*, which revealed no apparent correlation between seed coat color and population structure (Fig. S9). To uncover the underlying mechanism, we carried out a complementary association analysis using filtered SNPs, indels and SVs. A single association signal peak was observed in SV-GWAS, and a similar peak was identified in the same region in SNP-GWAS and indel-GWAS but with a high background (Fig. S8a-c). We then investigated the linkage disequilibrium extent based on SNPs, Indels and SVs in the Chr7:49–53 Mb and identified three independent blocks at 49.23–49.60 Mb, 49.74–51.70 Mb and 51.75–52.67 Mb (Fig. S8d-e). The most significant associated signals were from the second block of 1.96 Mb, including 174 SNPs, 693 indels and 71 SVs that were predicted to have disruptive impact on 26 out of 33 genes (Table S9). A candidate gene, *LG7_36701* that encodes a *bHLH* transcription factor homologous to the Arabidopsis *TT8*, was found the closest to the most significant signal (Chr7: 50,243,267, *P* = 1.80 × 10^–40^). In *A. thaliana*, the *tt8* knockout mutant displayed brown seed coat color [22], and therefore the lettuce homolog may play a similar role in anthocyanin biosynthesis in seed coat.

## Discussion

In this study, a total of 3,693,607 SVs were identified from 333 *L. sativa* and *L. serriola* accessions using an optimized pipeline. An in-depth comparison of SVs between wild and cultivated lettuce revealed the complexity of SVs in the lettuce genome.

SV identification is crucial to understand the genetic diversity within a given population and to investigate the impact of sequence variations [1, 23–25]. Whole genome sequencing (WGS) with short reads still represents a cost-effective way in the circumstance. Numerous SV detection software have been developed with WGS sequencing data based on read depth (RD), read pairs (RP) reads or split reads (SRs) [24, 26], and a great abundance of short-read sequence data accumulated in public databases for major crop species [27]. Because these software adopt different algorithms and perform differently in SV detection, it is recommended to merge outputs from multiple software to increase the accuracy [28]. However, this approach still has its limitations in detect complex SVs and resolve inconsistency in SV coordinates. Recently, long read sequencing enables SV detection from complex genomic regions and improving the detection rate of SV, and therefore facilitates graph-based pan-genome construction, for example in soybean, rice, tomato, and potato [29–32]. The graph-based pangenome construction and SV genotyping by WGS would provide a cost-effective way to detect SVs within large germplasm collections in the future.

GWAS with high-density SNPs is usually capable of identifying genomic regions associated with investigated traits, but without a comprehensive variant set it is often difficult to pinpoint causative mutations [33–35]. Recent studies demonstrate that including SVs in GWAS analyses explain additional heritability and increase the possibility for causal variant discovery [4, 30, 35, 36]. Association of SNP with lettuce leaf anthocyanin identified a 3.29-Mb region from 82.85–86.14 Mb on chromosome 5 [17], which enclosed the 84.53–86.16-Mb region detected by SV-GWAS. This demonstrates a strong association of *RLL2A* with leaf anthocyanin content detected by both SNPs and SVs. Recent work found SVs explained missing heritability by resolving incomplete LD, allelic heterogeneity, and locus heterogeneity in tomato [30]. The evidence from molecular and genetic studies in rice that SVs could cause major phenotypic variation. For example, an SV located on chromosome 3 (present and absent variant, 824 bp) may disrupt the formation of serine carboxypeptidase family proteins [37]. This protein plays a key role in the regulation of grain size [38]. Compared with the GWAS based on SNP, the SV-GWAS yields fewer significant associations, it could identify candidate regions for traits changes due to SVs [37, 39]. Therefore, utilizing a comprehensive variation set containing SVs for candidate interval identification enhances the probability of finding candidate genes and causal mutations, which suggest that future association studies would benefit from analysis using a comprehensive variation set.

## Conclusions

Here in this study, we generated a comprehensive SV set and characterized its distribution in the 333 *L. sativa* and *L. serriola* genebank accessions. Further association analyses revealed candidate variants and genes related to agronomic traits in cultivated lettuce. Overall, this work provides a valuable genomic resource for future lettuce research and breeding.

## Materials and methods

### SV identification

Raw sequencing data of 333 were downloaded from the CNGB Sequence Archive (CNSA) under the project ID CNP0000335 for 333 *Lactuca* accessions [17]. The raw data were generated in the BGISEQ-500 platform, and filtered by Trimmomatic (version 0.27) [40] with the parameters ILLUMINACLIP:adapter.fa:2:35:4:12:true LEADING:3 TRAILING:3 SLIDINGWINDOW:5:15 MINLEN:50. The filtered reads were mapped to the *L. sativa* cv. Salinas reference genome (version 8.0) [16] using BWA (version 0.7.12) [41] with the mem algorithm using the default parameters. Selected three widely-used SV callers, including Manta (version: 1.5.0.centos6_x86_64) [18], Delly (version: 0.7.8) [19], and Breakdancer (version: 1.3.6) [20] were run each accession to identify SVs. In order to improve detection accuracy, SVs were detected by at least two SV callers in our work, and those above 10 Mb were excluded from the data set. To check accuracy during SV merging, a total of manually confirmed non-redundant 229 SVs on chromosome 8 identified in a *L. serriola* accessions, TKI-340, were selected for manual authenticity validation manual curation (Table S10). The bam file was imported into the Integrative Genomics Viewer (IGV) [42] software, and the types and positions of the SVs within the region were manually validated based on read alignment (Fig. S10). In the same region, our SV calling procedure detected 142 SVs, including 135 overlapped with the reference SV set and seven false ones. Among the 55 SVs detected by all three software, only two SVs were more than 10 bp apart (18 bp and 41 bp) (Table S10). Based on 229 validated SVs, the maximum distance (--max_size_difference 10) for SV merging was set as the optimal parameter for svimmer (version 0.1) [43]. Then the SVs identified from each accession and from three software were merged into one vcf file using svimmer with the parameters ‘--max_distance 10 --max_size_difference 10’. The impacts of SVs that overlapped with the genic region or 1-kb flanking regions were assessed using the SnpEff (version 4.3r) [44] software with the parameter ‘-ud 1000’. The ‘-ud’ sets the size of upstream or downstream sequences.

### Comparison of SV frequencies

The frequencies of SVs in *L. sativa* and *L. serriola* were calculated within a filtered SV set with the minor allele frequency (MAF) of < 0.05. The significance of the differences in the SV frequencies between two groups was determined using the Fisher’s exact test, with the resulting *p* values corrected by the false discovery rate (FDR) method implemented in the R package “fdrtool”. SVs with FDR < 0.0001 and the ratio of allele frequency between *L. sativa* and *L. serriola* > 2 were classified as significantly differentially distributed. The protein-coding genes overlapped with differentially distributed SVs were used in an enrichment analysis of Gene Ontology (GO) and Kyoto Encyclopedia of Genes and Genomes (KEGG) pathway with the EnrichPipeline [45].

### Population structure analysis

A principal component analysis (PCA) was carried out on the filtered SV set using GCTA (version 1.91.4beta3) [46], and a neighbor-joining tree was constructed using PHYLIP (version 3.696) [47]. Population structure was deduced using Admixture (version 1.3.0) [48] with each *K* from 2 to 10 repeated 20 times, whose outputs were aligned by CLUMPP (version 1.1.2) [49].

### Genome-wide association of agronomic traits

Phenotypic records were downloaded from the Centre for Genetic Resources, the Netherlands (CGN) (http://www.wur.eu/cgnsc002) website (Table S11). Genome-wide association analyses were carried out on seven agronomic traits in *L. sativa*, including seed coat color, flower anthocyanin presence, leaf margin undulation, leaf anthocyanin content, seedling cotyledon shape, leaf venation and leaf morphology. A mixed linear regression model was run on the filtered SV set using EMMAX (beta-07Mar2010 version) [50] with five principal components and Balding–Nichols kinship [51] as covariance. For seed coat color, a filtered SNP and inset set was also used for GWAS using EMMAX. The SNPs and indels were filtered with a missing rate of > 10% or a minor allele frequency (MAF) of < 0.05, and the SNP set was further pruning by PLINK using a window size of 10 kb with a step size of one SNP and an *r*^2^ threshold of 0.5, as previously reported [17]. The significance threshold was adjusted using the Bonferroni method.

### Phylogenetic analysis of *RLL2*

To get the full sequences of gene *RLL2* in *L. sativa* germplasm, raw reads were assembled into contigs by SOAPdenovo (version 2.04; https://github.com/BGI-Qingdao/SOAPdenovoLR) for three accessions with high anthocyanin content, TKI-073, TKI-117 and TKI-122, and three with low content, TKI-001, TKI-074 and TKI-086. The *RLL2* alleles were retrieved by blastn [52]. A phylogenetic tree was constructed with the RLL2 protein sequences using MEGA (version 11) [53] by the maximum likelihood method.

To detect the large SVs around *RLL2*, we developed a custom script based on read alignment filles. Sequencing depth was calculated within a 100-kb window with 10-kb step using igvtools (Version 2.4.15) [54]. Deletions were determined as regions with its sequencing depth below half of the average depth across the genome, as implemented in CNVnator [55].

### Supplementary Information

**Additional file 1: Supplementary Table 1.** Variation statistics in each *Lactuca* accession. **Supplementary Table 2.** Summary of the structural variants and affected gene numbers. **Supplementary Table 3.** Summary of the structural variants and affected gene numbers in *L. sativa* and *L. serriola* with significant allele frequency differences. **Supplementary Table 4.** Enrichment of GO germ in the genes affected by *L. sativa*-predominant SVs. **Supplementary Table 5.** Enrichment of KEGG pathways in the genes affected by *L. sativa*-predominant SVs. **Supplementary Table 6.** Number of unique SVs in different geographic groups. **Supplementary Table 7.** Enrichment of KEGG pathways in the genes overlapping with SVs with significant allele frequency differences in different geographic group. **Supplementary Table 8.** Genomic regions associated with agronomic traits in the lettuce genome. **Supplementary Table 9.** Candidate genes associated with seed coat color. **Supplementary Table 10.** A total of 229 manually curated SVs using the Integrative Genomics Viewer (IGV) software to produce a reference set. **Supplementary Table 11.** Agronomic traits of 133 *L. sativa* accessions used for genome-wide association analyses.

**Additional file 2: Fig. S1.** Detection frequencies of SVs with different sizes. a. DEL; b. INS; c. DUP; d. INV. **Fig. S2.** SVs in 133 *L. sativa* accessions. a. The distribution of SVs along the lettuce genome. Circos plot from outer to inter tracks shows, (I) gene density in a 1-Mb sliding window, (II) GC content, (III-VII) SV count in the 1-Mb window for various SV types, including DEL (III), INS (IV), TRA (V), INV (VI), and DUP (VII). b. The number of different SV types. c. Detection frequencies of SVs with different sizes. d. The density of SVs and gene on each chromosome (count per Mb). e. The number of SVs from different generic regions. **Fig. S3.** SVs in 200 *L. serriola* accessions. a. The distribution of SVs along the lettuce genome. Circos plot from outer to inter tracks shows, (I) gene density in a 1-Mb sliding window, (II) GC content, (III-VII) SV count in the 1-Mb window for various SV types, including DEL (III), INS (IV), TRA (V), INV (VI), and DUP (VII). b. The number of different SV types. c. Detection frequencies of SVs with different sizes. d. The density of SVs and gene on each chromosome (count per Mb). e. The number of SVs from different generic regions. **Fig. S4.** Principal component analysis (PCA) using the filtered SVs of *L. sativa *(a) and* L. serriola *(b). **Fig. S5.** Cross-validation errors for each K from 1 to 10. Each box plot represents the values from 20 independent Admixture runs with randomly chosen seeds. The inner line represents the median, and the upper and lower bounds of the box represent the percentile of 25th and 75th, respectively. Whiskers represent 1.5 times of the interquartile range, and points outside the box are outliers. **Fig. S6.** Model-based clustering analysis with different numbers of ancestry kinship (*K*) from 2 to 10. Species are indicated in the colored bar at the bottom, with the green color for *L. sativa* and orange for *L. serriola*. Geographic groups of *L. serriola* are indicated in text from central Asia (CAS), the Caucasus (CAU), western Asia (WAS), southern Europe (SEU), and eastern Europe (EEU). Turkish samples and the admixed ones from *L. sativa* and *L. serriola* are indicated by arrows. **Fig. S7.** Genome-wide association results of leaf margin undulation (a), seedling cotyledon shape (b), leaf venation (c), leaf morphology (d), and flower anthocyanin presence (e), leaf anthocyanin content (f). Manhattan plots are shown in the left and Q-Q plots are in the right for each agronomic trait. **Fig. S8.** Identification of a *bHLH* gene associated with seed coat color in *L. sativa*. Manhattan plots of the genome-wide association results of seed coat color using SNPs (a), indels (b), and SVs (c). Manhattan plots are shown in the left and Q-Q plots are in the right for each data set. d. Manhattan plot of GWAS using SVs (red triangles), indels (blue dots), and SNPs (black dots) within the Chr7:49.23-52.92 Mb region. e. linkage disequilibrium (LD) heatmap using all the variants within the Chr7:49.23-52.92 Mb region filtered with MAF < 0.05. **Fig. S9.** Principal component analysis (PCA) using the filtered SNP (a), Indel (b) and SV(c) of *L. sativa*, in which black dots are represents seed coat color is black, white hollow dots represents seed coat color is white. **Fig. S10.** Read mapping from a *L. serriola* accession TKI-340. a-j. Overall view of 10 SVs with different coordinates detected by three SV calling software, from above Breakdancer, Delly, Manta.

**Additional file 3.**

## Data Availability

The data supporting this study are available in the supplementary material of this article. The raw data of this study is available at CNGB Nucleotide Sequence Archive (CNSA; the accession number CNP0000335).

## Abbreviations

SVs: Structural variations
SNPs: Single-nucleotide polymorphisms
Indels: Insertion/deletions
IGV: Integrative Genomics Viewer
GWAS: Genome-wide association study
MAF: Minor allele frequency
FDR: False discovery rate
GO: Gene Ontology
KEGG: Kyoto Encyclopedia of Genes and Genomes
PCA: A principal component analysis
CGN: The Centre for Genetic Resources, the Netherlands
DEL: Deletion
INS: Insertion
DUP: Duplication
TRA: Translocation
INV: Inversion
CAS: Central Asia
CAU: The Caucasus
WAS: Western Asia
SEU: Southern Europe
EEU: Eastern Europe
WGS: Whole genome sequencing
