# Supplementary material for: A comprehensive lettuce variation map reveals the impact of structural variations in agronomic traits
Source: BMC Genomics. 2023 Nov 2;24:659. doi: 10.1186/s12864-023-09739-x (PMC10621239; doi:10.1186/s12864-023-09739-x)
Supplement: Supplementary file 2 — Additional file 2: Fig. S1. Detection frequencies of SVs with different sizes. a. DEL; b. INS; c. DUP; d. INV. Fig. S2. SVs in 133 L. sativa accessions. a. The distribution of SVs along the lettuce genome. Circos plot from outer to inter tracks shows, (I) gene density in a 1-Mb sliding window, (II) GC content, (III-VII) SV count in the 1-Mb window for various SV types, including DEL (III), INS (IV), TRA (V), INV (VI), and DUP (VII). b. The number of different SV types. c. Detection frequencies of SVs with different sizes. d. The density of SVs and gene on each chromosome (count per Mb). e. The number of SVs from different generic regions. Fig. S3. SVs in 200 L. serriola accessions. a. The distribution of SVs along the lettuce genome. Circos plot from outer to inter tracks shows, (I) gene density in a 1-Mb sliding window, (II) GC content, (III-VII) SV count in the 1-Mb window for various SV types, including DEL (III), INS (IV), TRA (V), INV (VI), and DUP (VII). b. The number of different SV types. c. Detection frequencies of SVs with different sizes. d. The density of SVs and gene on each chromosome (count per Mb). e. The number of SVs from different generic regions. Fig. S4. Principal component analysis (PCA) using the filtered SVs of L. sativa (a) and L. serriola (b). Fig. S5. Cross-validation errors for each K from 1 to 10. Each box plot represents the values from 20 independent Admixture runs with randomly chosen seeds. The inner line represents the median, and the upper and lower bounds of the box represent the percentile of 25th and 75th, respectively. Whiskers represent 1.5 times of the interquartile range, and points outside the box are outliers. Fig. S6. Model-based clustering analysis with different numbers of ancestry kinship (K) from 2 to 10. Species are indicated in the colored bar at the bottom, with the green color for L. sativa and orange for L. serriola. Geographic groups of L. serriola are indicated in text from central Asia (CAS), the Caucasus (CAU [file 12864_2023_9739_MOESM2_ESM.docx]

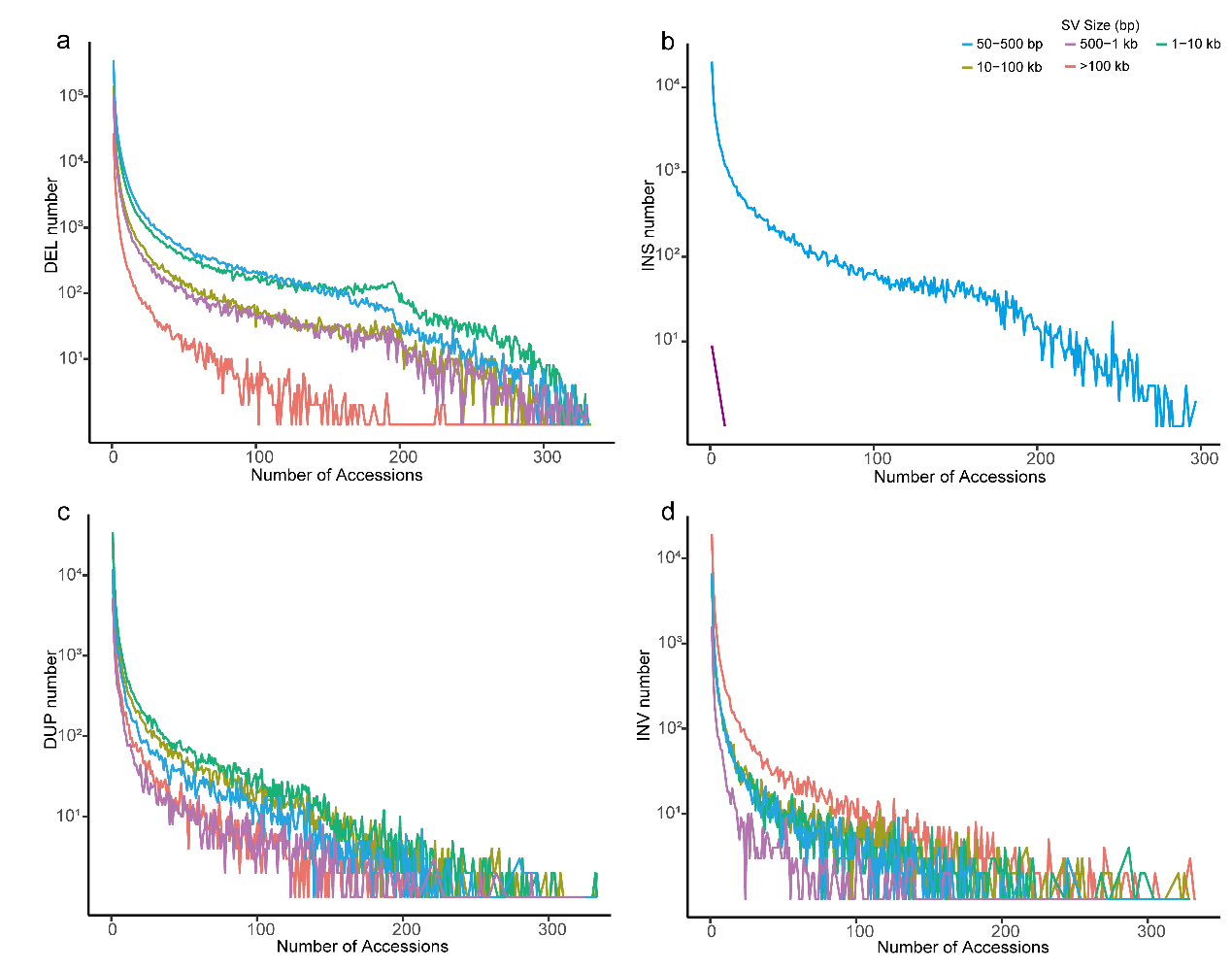


Fig. S1. Detection frequencies of SVs with different sizes. a. DEL; b. INS; c. DUP; d. INV.


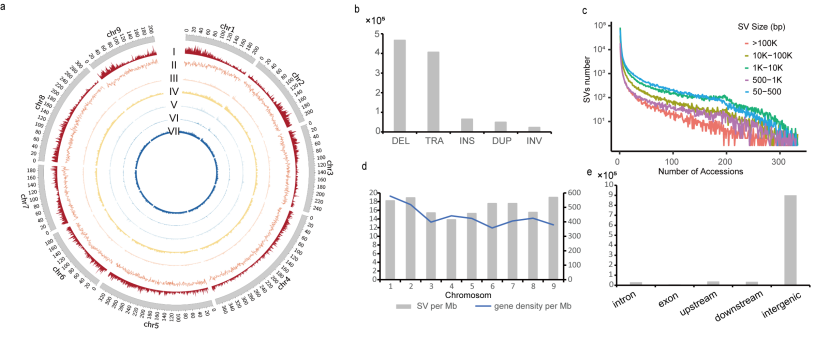


Fig. S2. SVs in 133 *L. sativa* accessions. **a**. The distribution of SVs along the lettuce genome. Circos plot from outer to inter tracks shows, (I) gene density in a 1-Mb sliding window, (II) GC content, (III-VII) SV count in the 1-Mb window for various SV types, including DEL (III), INS (IV), TRA (V), INV (VI), and DUP (VII). **b.** The number of different SV types. **c.** Detection frequencies of SVs with different sizes. **d.** The density of SVs and gene on each chromosome (count per Mb). **e.** The number of SVs from different generic regions.


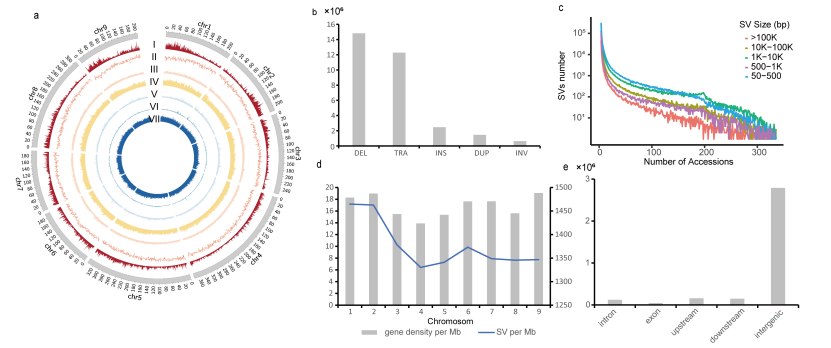


Fig. S3. SVs in 200 *L. serriola* accessions. **a**. The distribution of SVs along the lettuce genome. Circos plot from outer to inter tracks shows, (I) gene density in a 1-Mb sliding window, (II) GC content, (III-VII) SV count in the 1-Mb window for various SV types, including DEL (III), INS (IV), TRA (V), INV (VI), and DUP (VII). **b.** The number of different SV types. **c.** Detection frequencies of SVs with different sizes. **d.** The density of SVs and gene on each chromosome (count per Mb). **e.** The number of SVs from different generic regions.


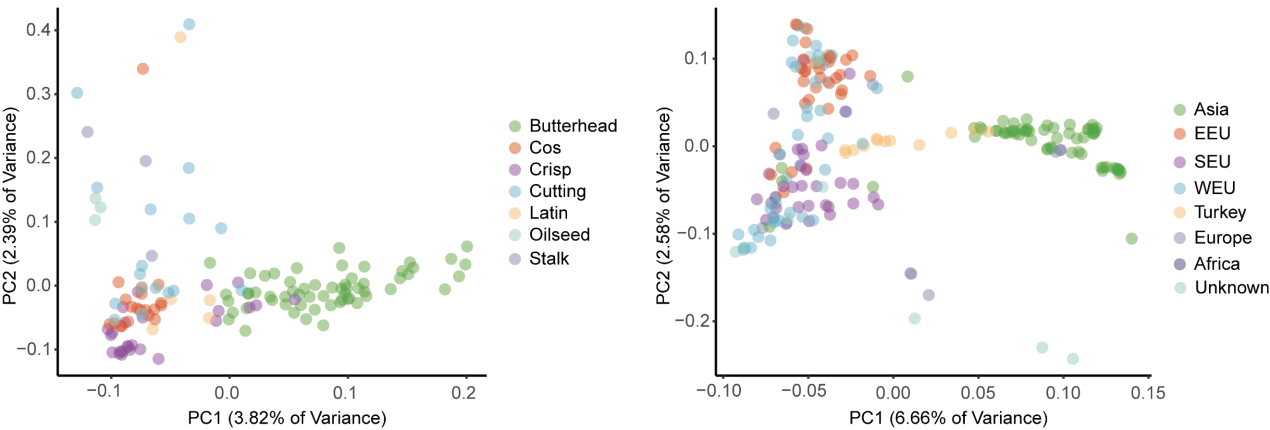


Fig. S4. Principal component analysis (PCA) using the filtered SVs of *L. sativa* (a) and *L. serriola* (b)*.*


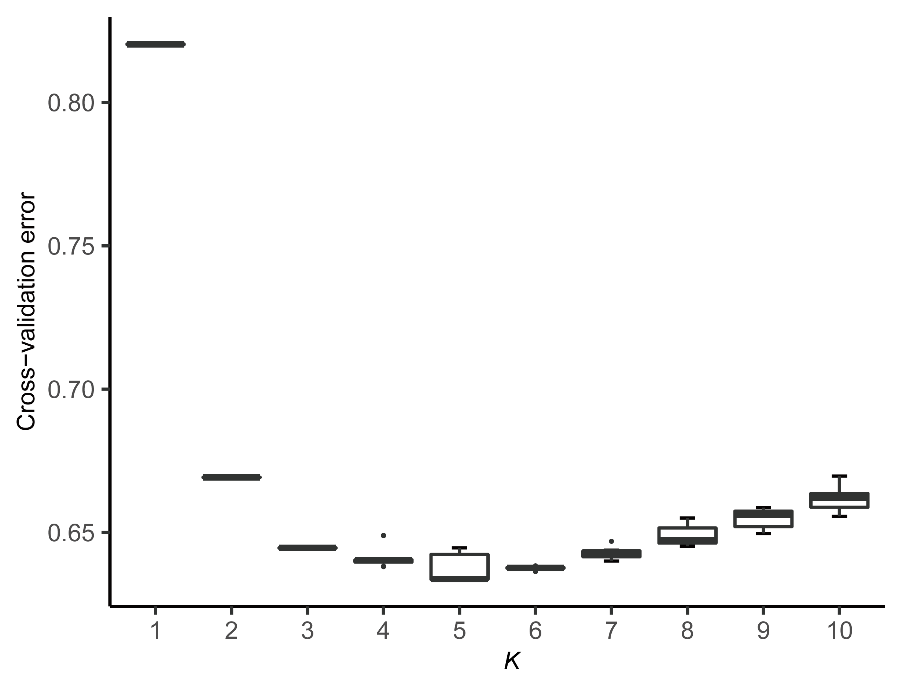


Fig. S5. Cross-validation errors for each K from 1 to 10. Each box plot represents the values from 20 independent Admixture runs with randomly chosen seeds. The inner line represents the median, and the upper and lower bounds of the box represent the percentile of 25th and 75th, respectively. Whiskers represent 1.5 times of the interquartile range, and points outside the box are outliers.


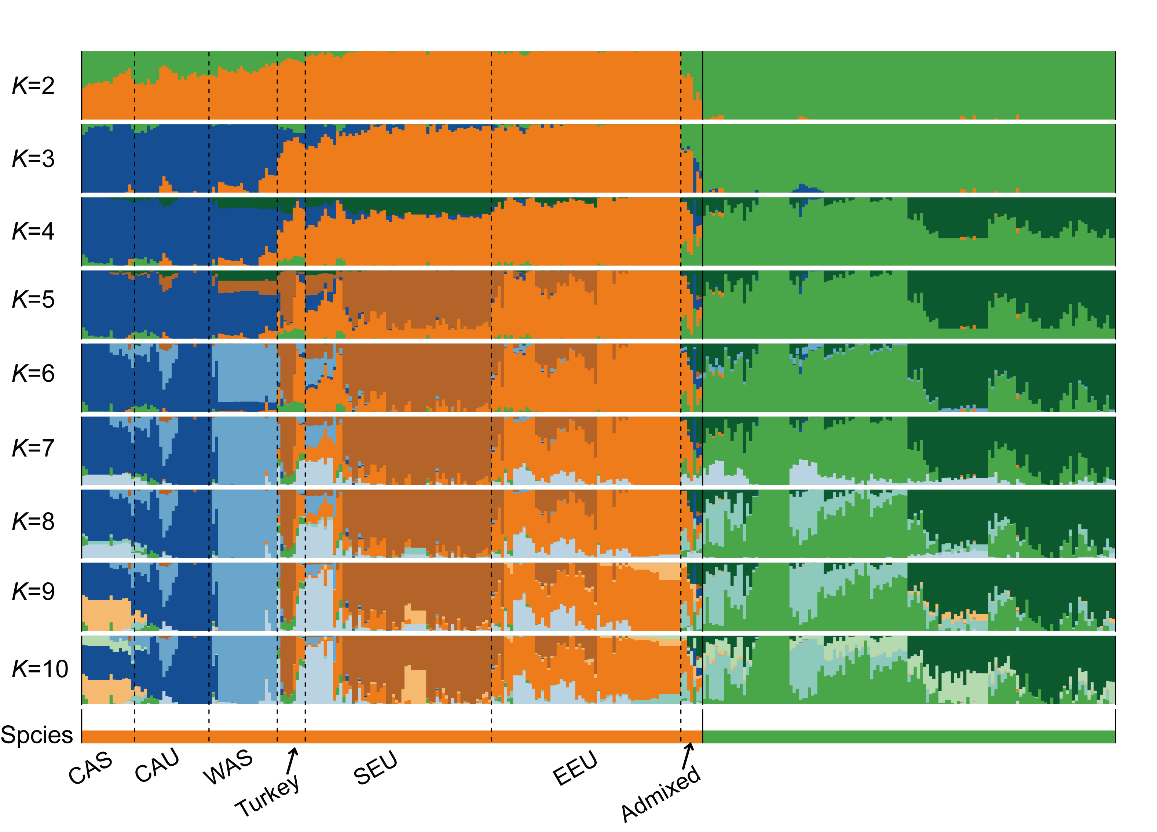


Fig. S6. Model-based clustering analysis with different numbers of ancestry kinship (*K*) from 2 to 10. Species are indicated in the colored bar at the bottom, with the green color for *L. sativa* and orange for *L. serriola*. Geographic groups of *L. serriola* are indicated in text from central Asia (CAS), the Caucasus (CAU), western Asia (WAS), southern Europe (SEU), and eastern Europe (EEU). Turkish samples and the admixed ones from *L. sativa* and *L. serriola* are indicated by arrows.


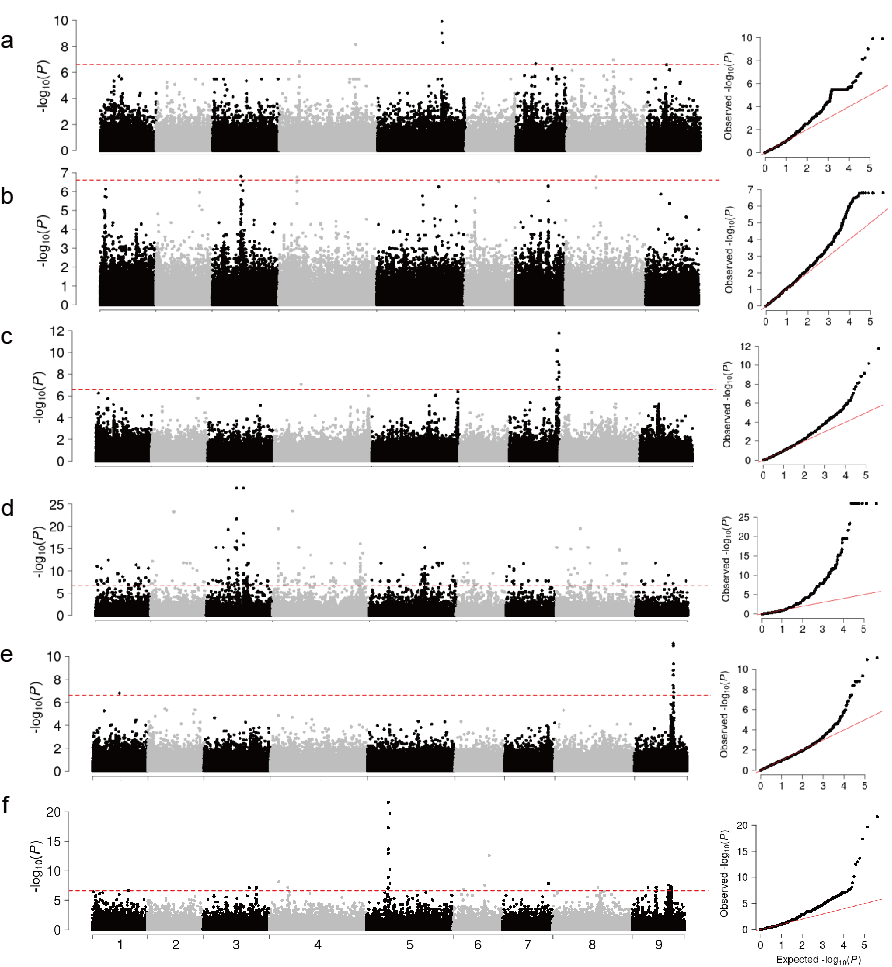


Fig. S7. Genome-wide association results of leaf margin undulation (**a**), seedling cotyledon shape (**b**), leaf venation (**c**), leaf morphology (**d**), and flower anthocyanin presence (**e**), leaf anthocyanin content (**f**). Manhattan plots are shown in the left and Q-Q plots are in the right for each agronomic trait.


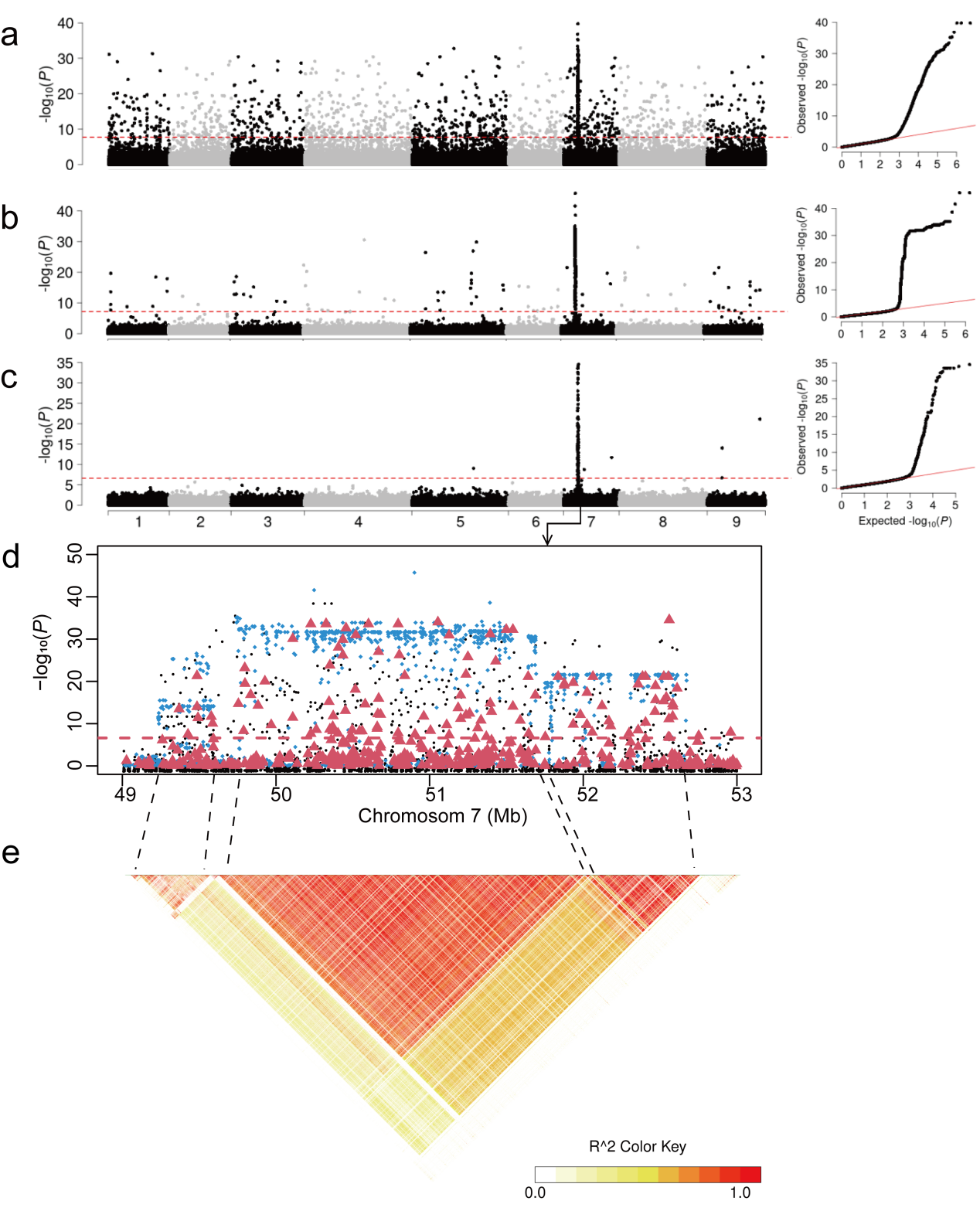


Fig. S8. Identification of a *bHLH* gene associated with seed coat color in *L. sativa*. Manhattan plots of the genome-wide association results of seed coat color using SNPs (**a**)，indels (**b**), and SVs (**c**). Manhattan plots are shown in the left and Q-Q plots are in the right for each data set. **d**. Manhattan plot of GWAS using SVs (red triangles), indels (blue dots), and SNPs (black dots) within the Chr7:49.23-52.92 Mb region. **e**. linkage disequilibrium (LD) heatmap using all the variants within the Chr7:49.23-52.92 Mb region filtered with MAF < 0.05.


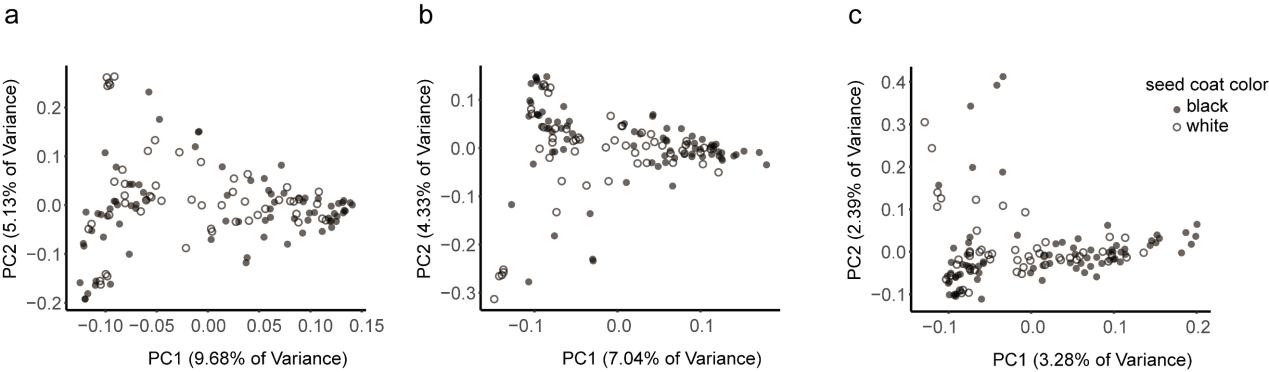


Fig. S9. Principal component analysis (PCA) using the filtered SNP (**a**), Indel (**b**) and SV(**c**) of *L. sativa*, in which black dots are represents seed coat color is black, white hollow dots represents seed coat color is white.


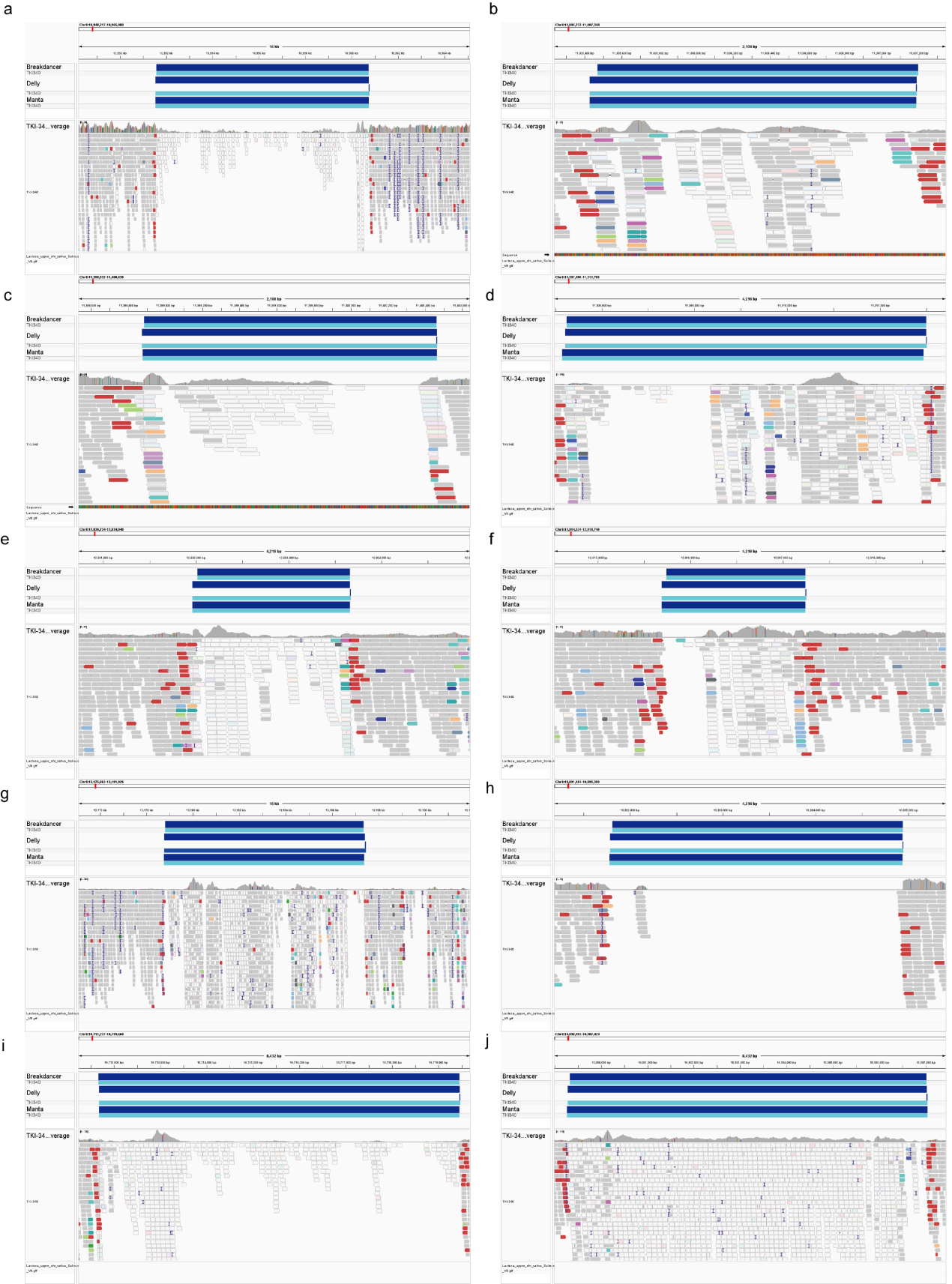


Fig. S10. Read mapping from a *L. serriola* accession TKI-340. a-j. Overall view of 10 SVs with different coordinates detected by three SV calling software, from above Breakdancer, Delly, Manta.
