# Supplementary material for: A comprehensive lettuce variation map reveals the impact of structural variations in agronomic traits
Source: BMC Genomics. 2023 Nov 2;24:659. doi: 10.1186/s12864-023-09739-x (PMC10621239; doi:10.1186/s12864-023-09739-x)
Supplement: Supplementary file 3 — Additional file 3. [file 12864_2023_9739_MOESM3_ESM.docx]

>TKI-001_RLL2B-Y37

ATGACACCAAGCAGTAGTAATGGTTTAGGATTGAAGAAAGGTGCATGGAGTGCACATGAAGATAAGCTTC

TCAAGGATTATATTGAACAATACGGCGAAGGGAAATGGCACCTTATTCCTCGCAGAACCGGGTTAAATAG

ATGTAGAAAGAGTTGCAGGCTACGATGGTTGAATTATTTGAGGCCAAATATAAAACGAGGTGATTTTGCT

GAAGATGAAGTTGATCTCATGCTTAGGCTCCATCAGATTCTAGGAAACAGGTGGTCGTTAATTGCGGGAA

GAATACCTGGAAGAACTGCTAATGACGTGAAGAATTATTGGAACACTCATATTCAGCCTCGTTCCAAACA

ACAAAAGACAGAACTGTTACAAGACGCTCCAGTCACAAGTATCAATCCACAACCGCATACCATGTCCAAA

ACCCAAAATTTGTCCACGAGTGACAAAACACAAATTGTCTCCAATGGTGGTGGTAATTTTATAAGCTCGT

CGAATGAAGGTGGCAACAACAACTTTAACGTCTCCTCTCTGTCATCAAATGTGCTACATGATGACAAGAT

TAAAAAATATCTTGACGAATTATTTGATGATCGTGAAATGGAAATTGAAGGAGACATTGAATGGTCATTT

GGTGAATCTTCAGCGCAGGCAGAGGCCTTAGATGTTGTCGATCAAGAAGAAGAGAACAATGGTTTCTTTG

ATTTCTCGCTGGATGAGATCATGTTGAACCCAATGGATTCACAGCAACCTTGA

>TKI-082_RLL2B-Y37

ATGACACCAAGCAGTAGTAATGGTTTAGGATTGAAGAAAGGTGCATGGAGTGCACATGAAGATAAGCTTC

TCAAGGATTATATTGAACAATACGGCGAAGGGAAATGGCACCTTATTCCTCGCAGAACCGGGTTAAATAG

ATGTAGAAAGAGTTGCAGGCTACGATGGTTGAATTATTTGAGGCCAAATATAAAACGAGGTGATTTTGCT

GAAGATGAAGTTGATCTCATGCTTAGGCTCCATCAGATTCTAGGAAACAGGTGGTCGTTAATTGCGGGAA

GAATACCTGGAAGAACTGCTAATGACGTGAAGAATTATTGGAACACTCATATTCAGCCTCGTTCCAAACA

ACAAAAGACAGAACTGTTACAAGACGCTCCAGTCACAAGTATCAATCCACAACCGCATACCATGTCCAAA

ACCCAAAATTTGTCCACGAGTGACAAAACACAAATTGTCTCCAATGGTGGTGGTAATTTTATAAGCTCGT

CGAATGAAGGTGGCAACAACAACTTTAACGTCTCCTCTCTGTCATCAAATGTGCTACATGATGACAAGAT

TAAAAAATATCTTGACGAATTATTTGATGATCGTGAAATGGAAATTGAAGGAGACATTGAATGGTCATTT

GGTGAATCTTCAGCGCAGGCAGAGGCCTTAGATGTTGTCGATCAAGAAGAAGAGAACAATGGTTTCTTTG

ATTTCTCGCTGGATGAGATCATGTTGAACCCAATGGATTCACAGCAACCTTGA

>TKI-094_RLL2B-Y37

ATGACACCAAGCAGTAGTAATGGTTTAGGATTGAAGAAAGGTGCATGGAGTGCACATGAAGATAAGCTTC

TCAAGGATTATATTGAACAATACGGCGAAGGGAAATGGCACCTTATTCCTCGCAGAACCGGGTTAAATAG

ATGTAGAAAGAGTTGCAGGCTACGATGGTTGAATTATTTGAGGCCAAATATAAAACGAGGTGATTTTGCT

GAAGATGAAGTTGATCTCATGCTTAGGCTCCATCAGATTCTAGGAAACAGGTGGTCGTTAATTGCGGGAA

GAATACCTGGAAGAACTGCTAATGACGTGAAGAATTATTGGAACACTCATATTCAGCCTCGTTCCAAACA

ACAAAAGACAGAACTGTTACAAGACGCTCCAGTCACAAGTATCAATCCACAACCGCATACCATGTCCAAA

ACCCAAAATTTGTCCACGAGTGACAAAACACAAATTGTCTCCAATGGTGGTGGTAATTTTATAAGCTCGT

CGAATGAAGGTGGCAACAACAACTTTAACGTCTCCTCTCTGTCATCAAATGTGCTACATGATGACAAGAT

TAAAAAATATCTTGACGAATTATTTGATGATCGTGAAATGGAAATTGAAGGAGACATTGAATGGTCATTT

GGTGAATCTTCAGCGCAGGCAGAGGCCTTAGATGTTGTCGATCAAGAAGAAGAGAACAATGGTTTCTTTG

ATTTCTCGCTGGATGAGATCATGTTGAACCCAATGGATTCACAGCAACCTTGA

>TKI-081_RLL2A

ATGACATCGCACAGCCACAGTAGTAGTGGTTTAGGGTTGAAGAAAGGTGCATGGAGTGCACATGAAGATATGCTTCTCAAGAATTGTATTGAGCAATACGGCGAAGGGAAATGGCACCTTATTCCTGGCAGAACAGGTTTAAACAGATGTAGAAAGAGTTGTAGGCTACGATGGTTAAATTATCTAAGACCAAATATAAAAAGAGGTGATTTTGGTGAAGATGAAGTTGATCTCATGCTTAGGCTTCACAAGCTATTAGGGAACAGGTGGTCGTTAATTGCGGGAAGAATACCTGGAAGAACTGCTAATGACGTGAAGAATTATTGGAACACTCATATTTACTCAAAATCTGGTTTCAGTTAATCCACATTCGGATCCGATTTGGATCCAAATCATTTAACCGGTTTGCATGTGAGCCTTTAGATAGAATACTATTAATATATAATAGTATTATTTGTTTTCTTTAGCTTCCGACTTCGGTAACCTTTTATCTCTTAAAGCTCCGAATGTGTGGATTTGTTTTTGTGGAAATATGATTGTGCACGTAAGCATATATTTATGTGGACGGGGTGGTTGTTAAAAACATGCATGTATTTCTCTTTGTGTCTATAAAGGCGCATGGGTTCAGTGTAATTAATTCTATTCAAAGATTAATTATTAAATGAGCAAGTTCTGTTACTATAGAAAAAGAAGTAGAATTTCAACTTCAACCTTGTATGTATGTCCAACAACACTTGCATAAACTACAAATAAAATGAGACCAGGTAATAATAC

>TKI-125_RLL2A

ATGACATCGCACAGCCACAGTAGTAGTGGTTTAGGGTTGAAGAAAGGTGCATGGAGTGCACATGAAGATATGCTTCTCAAGAATTGTATTGAGCAATACGGCGAAGGGAAATGGCACCTTATTCCTGGCAGAACAGGAATAGATGTAGAAAGAGTTGTAGGCTACGATGGTTGAATTATCTGAGGCCAAATATAAAACGTGGTGATTTTGCTGAAGATGAAGTTGATCTCATGCTTAGGCTTCATAAACTTCTAGGAAACAGGTGGTCGTTAATTGCGGGAAGAATACCTGGAAGAACTGCTAATGACGTGAAGAATTATTGGAACACTCATATTCAGCCTCGTTCCAAACAACAAAAGATAGAACCTGATGCTGATGAACTGTTACAAGACGCTCCAGTCACAATTATACATCCACAACAGTATACCACCATGTCCAAAACCCAAAATTTGTCCACGAGTGACAAAACACAAATTGTCTCCAATGGTGGTGGTAATCTTATAAGCTCGTTGAATGAAGGTGGCAACAACTTTAACGTCTCCTCTCTATCATCTAATGTACTACTTGATGACAAGATTAAAAAATATCTTGACGAATTATTTGATGATCGTGAAATGGAAATTGAAGGAGACATTGAATGGTCATTTGGTGAATCTTCAGCGCAGGCAGAGGCCTTAGATGTTGTCGATCAAGAAGAAGATAACAATCGTTTCTTTGATTACTCGCTTGATGAGATCATGTGGAACCCAATGGATTCACAGCAACCATGA

>TKI-130_RLL2A

ATGACATCGCACAGCCACAGTAGTAGTGGTTTAGGGTTGAAGAAAGGTGCATGGAGTGCACATGAAGATATGCTTCTCAAGAATTGTATTGAGCAATACGGCGAAGGGAAATGGCACCTTATTCCTGGCAGAACAGGTTAAACAGATGTAGAAAGAGTTGTAGGCTACGATGGTTAAATTATCTAAGACCAAATATAAAAAGAGGTGATTTTGGTGAAGATGAAGTTGATCTCATGCTTAGGCTTCACAAGCTATTAGGGAACAGGTGGTCGTTAATTGCGGGAAGAATACCTGGAAGAACTGCTAATGACGTGAAGAATTATTGGAACACTCATATTCAGCCTCGTTCCAAACAACAAAAGATAGAACCTGATGCTGATGAACTGTTACAAGACGCTCCAGTCACAATTATACATCCACAACAGTATACCACCATGTCCAAAACCCAAAATTTGTCCACGAGTGACAAAACACAAATTGTCTCCAATGGTGGTGGTAATCTTATAAGCTCGTTGAATGAAGGTGGCAACAACTTTAACGTCTCCTCTCTATCATCTAATGTACTACTTGATGACAAGATTAAAAAATATCTTGACGAATTATTTGATGATCGTGAAATGGAAATTGAAGGAGACATTGAATGGTCATTTGGTGAATCTTCAGCGCAGGCAGAGGCCTTAGATGTTGTCGATCAAGAAGAAGATAACAATCGTTTCTTTGATTACTCGCTTGATGAGATCATGTGGAACCCAATGGATTCACAGCAACCATGA

>TKI-081_RLL2B

CCAGAACTTGAAATTCACGTTTATATATATTTACGGATAACATTCGCATAATTAGCACAAAAGAAATGACACCAAGCAGTAGTAATGCTTTAGGATTGAAGAAAGGTGCATGGAGTGCACATGAAGATAAGCTTCTCAAGGATTATATTGAGCAATACGGCGAAGGGAACTGGCATCTTATTCCTCGCAGAACCGGGTTAAATTGATATAGAAAGAGTTGCAGGAGACTACGATGGTTGAATTATTTGAGGCCAAATTTAAAACGAGGTGATTTTACTGAAGATGAAGTTGATCTCATGCTTAGGCTCCATAAGCTTCTAGGAAACAGTGGTCGTTAATTGCGGGAAGAATACCTGGAAGAACTGCTAATGACGTGAAGAATTATTGGAACACTCATATTCAGCCTCGTTCCAAACAACAAAAGATAGAACCTGATGCTGATGAACTGTTACAAGACGCTCCAGTCACAATTATACATCCACAACAGTATACCACCATGTCCAAAACCCAAAATTTGTCCACGAGTGACAAAACACAAATTGTCTCCAATGGTGGTGGTAATCTTATAAGCTCGTTGAATGAAGGTGGCAACAACTTTAACGTCTCCTCTCTATCATCTAATGTACTACTTGATGACAAGATTAAAAAATATCTTGACGAATTATTTGATGATCGTGAAATGGAAATTGAAGGAGACATTGAATGGTCATTTGGTGAATCTTCAGCGCAGGCAGAGGCCTTAGATGTTGTCGATCAAGAAGAAGATAACAATCGTTTCTTTGATTACTCGCTTGATGAGATCATGTGGAACCCAATGGATTCACAGCAACCATGATTTAACTTCGTTTCACTCATCGCCATGCATGTAATAAACCGAATAAATTAAAAAAGTAAGTATCGTGTCTATCAAATATTATATTGAATGTTTTCATGTAATATATCTA

>TKI-125_RLL2B

CCAGAACTTGAAATTCACGTTTATATATATTTACGGATAACATTCGCATAATTAGCACAAAAGAAATGACACCAAGCAGTAGTAATGCTTTAGGATTGAAGAAAGGTGCATGGAGTGCACATGAAGATAAGCTTCTCAAGGATTATATTGAGCAATACGGCGAAGGGAACTGGCATCTTATTCCTCGCAGAACCGGGTTAAATAGATGTAGAAAGAGTTGCAGGCTACGATGGTTGAATTATTTGAGGCCAAATATAAAACGAGGTGATTTTGCTGAAGATGAAGTTGATCTCATGCTTAGGCTCCATAAGATTCTAGGAAACAGGTGGTCGTTAATTGCGGGAAGAATACCTGGAAGAACTGCTAATGACGTGAAGAATTATTGGAACACTCATATTCAGCCTCGTTCCAAACAACAAAAGATAGAACCTGATGCTGATGAACTGTTACAAGACGCTCCAGTCACAATTATACATCCACAACAGTATACCACCATGTCCAAAACCCAAAATTTGTCCACGAGTGACAAAACACAAATTGTCTCCAATGGTGGTGGTAATCTTATAAGCTCGTTGAATGAAGGTGGCAACAACTTTAACGTCTCCTCTCTATCATCTAATGTACTACTTGATGACAAGATTAAAAAATATCTTGACGAATTATTTGATGATCGTGAAATGGAAATTGAAGGAGACATTGAATGGTCATTTGGTGAATCTTCAGCGCAGGCAGAGGCCTTAGATGTTGTCGATCAAGAAGAAGATAACAATCGTTTCTTTGATTACTCGCTTGATGAGATCATGTGGAACCCAATGGATTCACAGCAACCATGATTTAACTTCGTTTCACTCATCGCCATGCATGTAATAAACCGAATAAATTAAAAAAGTAAGTATCGTGTCTATCAAATATTATATTGAATGTTTTCATGTAATATATCTA

>TKI-130_RLL2B

CCAGAACTTGAAATTCACGTTTATATATATTTACGGATAACATTCGCATAATTAGCACAAAAGAAATGACACCAAGCAGTAGTAATGCTTTAGGATTGAAGAAAGGTGCATGGAGTGCACATGAAGATAAGCTTCTCAAGGATTATATTGAGCAATACGGCGAAGGGAACTGGCATCTTATTCCTCGCAGAACCGGGTTAAATAGATGTAGAAAGAGTTGCAGGCTACGATGGTTGAATTATTTGAGGCCAAATATAAAACGAGGTGATTTTGCTGAAGATGAAGTTGATCTCATGCTTAGGCTCCATAAGATTCTAGGAAACAGGTGGTCGTTAATTGCGGGAAGAATACCTGGAAGAACTGCTAATGACGTGAAGAATTATTGGAACACTCATATTCAGCCTCGTTCCAAACAACAAAAGATAGAACCTGATGCTGATGAACTGTTACAAGACGCTCCAGTCACAATTATACATCCACAACAGTATACCACCATGTCCAAAACCCAAAATTTGTCCACGAGTGACAAAACACAAATTGTCTCCAATGGTGGTGGTAATCTTATAAGCTCGTTGAATGAAGGTGGCAACAACTTTAACGTCTCCTCTCTATCATCTAATGTACTACTTGATGACAAGATTAAAAAATATCTTGACGAATTATTTGATGATCGTGAAATGGAAATTGAAGGAGACATTGAATGGTCATTTGGTGAATCTTCAGCGCAGGCAGAGGCCTTAGATGTTGTCGATCAAGAAGAAGATAACAATCGTTTCTTTGATTACTCGCTTGATGAGATCATGTGGAACCCAATGGATTCACAGCAACCATGATTTAACTTCGTTTCACTCATCGCCATGCATGTAATAAACCGAATAAATTAAAAAAGTAAGTATCGTGTCTATCAAATATTATATTGAATGTTTTCATGTAATATATCTA
